# Supplementary material for: Time-series transcriptome analysis identified differentially expressed genes in broiler chicken infected with mixed Eimeria species
Source: Front Genet. 2022 Aug 8;13:886781. doi: 10.3389/fgene.2022.886781 (PMC9393255; doi:10.3389/fgene.2022.886781)
Supplement: Supplementary file 2 [file DataSheet1.ZIP › 7dpi_GO.Gsea.1625106495923/GOBP_RESPIRATORY_ELECTRON_TRANSPORT_CHAIN.html]

Details for gene set GOBP\_RESPIRATORY\_ELECTRON\_TRANSPORT\_CHAIN[GSEA]

|  || Dataset | TMM\_7dpi\_gct\_format\_7dpi\_gct\_format.Class\_4dpi.cls #PC\_versus\_NC.Class\_4dpi.cls #PC\_versus\_NC\_repos |
| Phenotype | Class\_4dpi.cls#PC\_versus\_NC\_repos |
| Upregulated in class | 1 |
| GeneSet | GOBP\_RESPIRATORY\_ELECTRON\_TRANSPORT\_CHAIN |
| Enrichment Score (ES) | 0.719721 |
| Normalized Enrichment Score (NES) | 3.5252173 |
| Nominal p-value | 0.0 |
| FDR q-value | 0.0 |
| FWER p-Value | 0.0 |
Table: GSEA Results Summary

  

Fig 1: Enrichment plot: GOBP\_RESPIRATORY\_ELECTRON\_TRANSPORT\_CHAIN      
 Profile of the Running ES Score & Positions of GeneSet Members on the Rank Ordered List

  

| SYMBOL | TITLE | RANK IN GENE LIST | RANK METRIC SCORE | RUNNING ES | CORE ENRICHMENT || 1 | SCO2 | na | 194 | 1.052 | 0.0095 | Yes |
| 2 | DGUOK | na | 238 | 1.001 | 0.0304 | Yes |
| 3 | COX5A | na | 300 | 0.942 | 0.0483 | Yes |
| 4 | NDUFA2 | na | 303 | 0.935 | 0.0710 | Yes |
| 5 | NDUFB6 | na | 307 | 0.933 | 0.0936 | Yes |
| 6 | NDUFS5 | na | 308 | 0.932 | 0.1164 | Yes |
| 7 | NDUFB7 | na | 323 | 0.915 | 0.1377 | Yes |
| 8 | PPARGC1A | na | 339 | 0.894 | 0.1583 | Yes |
| 9 | NDUFB2 | na | 348 | 0.885 | 0.1793 | Yes |
| 10 | NDUFAB1 | na | 356 | 0.876 | 0.2001 | Yes |
| 11 | NDUFA7 | na | 371 | 0.857 | 0.2199 | Yes |
| 12 | NDUFC2 | na | 394 | 0.834 | 0.2385 | Yes |
| 13 | UQCR10 | na | 444 | 0.785 | 0.2536 | Yes |
| 14 | CYCS | na | 475 | 0.763 | 0.2697 | Yes |
| 15 | COX6A1 | na | 480 | 0.760 | 0.2880 | Yes |
| 16 | UQCR11 | na | 485 | 0.754 | 0.3061 | Yes |
| 17 | UQCRB | na | 487 | 0.753 | 0.3245 | Yes |
| 18 | NDUFA11 | na | 497 | 0.746 | 0.3420 | Yes |
| 19 | NDUFA13 | na | 557 | 0.708 | 0.3544 | Yes |
| 20 | NDUFS3 | na | 623 | 0.675 | 0.3654 | Yes |
| 21 | NDUFB3 | na | 656 | 0.657 | 0.3788 | Yes |
| 22 | NDUFS8 | na | 661 | 0.653 | 0.3945 | Yes |
| 23 | NDUFS6 | na | 686 | 0.642 | 0.4082 | Yes |
| 24 | SDHD | na | 708 | 0.632 | 0.4219 | Yes |
| 25 | UQCC3 | na | 735 | 0.620 | 0.4349 | Yes |
| 26 | NDUFA1 | na | 742 | 0.616 | 0.4494 | Yes |
| 27 | UQCRHL | na | 788 | 0.594 | 0.4602 | Yes |
| 28 | NDUFA5 | na | 794 | 0.593 | 0.4743 | Yes |
| 29 | SLC25A13 | na | 812 | 0.586 | 0.4872 | Yes |
| 30 | SDHB | na | 840 | 0.575 | 0.4990 | Yes |
| 31 | GHITM | na | 842 | 0.575 | 0.5130 | Yes |
| 32 | DNAJC15 | na | 863 | 0.566 | 0.5252 | Yes |
| 33 | NDUFB5 | na | 902 | 0.554 | 0.5356 | Yes |
| 34 | SDHAF2 | na | 919 | 0.550 | 0.5477 | Yes |
| 35 | COX6C | na | 930 | 0.546 | 0.5602 | Yes |
| 36 | COQ9 | na | 939 | 0.541 | 0.5728 | Yes |
| 37 | NDUFV3 | na | 988 | 0.527 | 0.5817 | Yes |
| 38 | NDUFB4 | na | 1015 | 0.517 | 0.5921 | Yes |
| 39 | NDUFA12 | na | 1023 | 0.513 | 0.6041 | Yes |
| 40 | COX7B | na | 1055 | 0.501 | 0.6138 | Yes |
| 41 | NDUFA6 | na | 1079 | 0.492 | 0.6239 | Yes |
| 42 | NDUFA8 | na | 1080 | 0.492 | 0.6359 | Yes |
| 43 | UQCRQ | na | 1103 | 0.484 | 0.6459 | Yes |
| 44 | NDUFS2 | na | 1124 | 0.477 | 0.6559 | Yes |
| 45 | COX4I1 | na | 1125 | 0.476 | 0.6676 | Yes |
| 46 | SDHC | na | 1135 | 0.473 | 0.6784 | Yes |
| 47 | NDUFB10 | na | 1215 | 0.450 | 0.6828 | Yes |
| 48 | UQCRFS1 | na | 1263 | 0.437 | 0.6896 | Yes |
| 49 | NDUFV1 | na | 1307 | 0.428 | 0.6964 | Yes |
| 50 | NDUFB8 | na | 1330 | 0.423 | 0.7049 | Yes |
| 51 | NDUFS7 | na | 1371 | 0.412 | 0.7117 | Yes |
| 52 | NDUFV2 | na | 1395 | 0.408 | 0.7197 | Yes |
| 53 | BID | na | 1694 | 0.334 | 0.7029 | No |
| 54 | UQCRC2 | na | 1700 | 0.332 | 0.7106 | No |
| 55 | COX7A2L | na | 1761 | 0.316 | 0.7133 | No |
| 56 | NDUFB9 | na | 1842 | 0.299 | 0.7139 | No |
| 57 | COX15 | na | 1927 | 0.282 | 0.7138 | No |
| 58 | NDUFB1 | na | 1995 | 0.272 | 0.7148 | No |
| 59 | ETFA | na | 2069 | 0.259 | 0.7151 | No |
| 60 | TAZ | na | 2298 | 0.223 | 0.7014 | No |
| 61 | UQCRC1 | na | 2326 | 0.217 | 0.7044 | No |
| 62 | NDUFA4 | na | 2726 | 0.160 | 0.6749 | No |
| 63 | NDUFA9 | na | 2879 | 0.141 | 0.6656 | No |
| 64 | ISCU | na | 2900 | 0.138 | 0.6673 | No |
| 65 | COX10 | na | 3408 | 0.073 | 0.6266 | No |
| 66 | NDUFA10 | na | 3412 | 0.073 | 0.6281 | No |
| 67 | SDHA | na | 3488 | 0.064 | 0.6234 | No |
| 68 | NDUFS1 | na | 3763 | 0.033 | 0.6012 | No |
| 69 | PARK7 | na | 3768 | 0.032 | 0.6017 | No |
| 70 | NDUFAF1 | na | 3921 | 0.014 | 0.5893 | No |
| 71 | SLC25A12 | na | 4116 | -0.008 | 0.5732 | No |
| 72 | CDK1 | na | 4341 | -0.035 | 0.5553 | No |
| 73 | NDUFS4 | na | 4402 | -0.040 | 0.5512 | No |
| 74 | COX7C | na | 4881 | -0.090 | 0.5134 | No |
| 75 | SNCA | na | 5117 | -0.114 | 0.4965 | No |
| 76 | SLC25A22 | na | 5176 | -0.118 | 0.4945 | No |
| 77 | ETFRF1 | na | 5387 | -0.138 | 0.4803 | No |
| 78 | DLD | na | 5596 | -0.158 | 0.4667 | No |
| 79 | MYBBP1A | na | 5686 | -0.168 | 0.4633 | No |
| 80 | AIFM2 | na | 6160 | -0.220 | 0.4291 | No |
| 81 | GPD2 | na | 6355 | -0.239 | 0.4186 | No |
| 82 | IMMP2L | na | 6710 | -0.275 | 0.3957 | No |
| 83 | ETFDH | na | 7278 | -0.338 | 0.3564 | No |
| 84 | CCNB1 | na | 8681 | -0.511 | 0.2514 | No |
| 85 | POLG2 | na | 11536 | -1.131 | 0.0397 | No |
Table: GSEA details [plain text format]

  

Fig 2: GOBP\_RESPIRATORY\_ELECTRON\_TRANSPORT\_CHAIN      
 Blue-Pink O' Gram in the Space of the Analyzed GeneSet

  

Fig 3: GOBP\_RESPIRATORY\_ELECTRON\_TRANSPORT\_CHAIN: Random ES distribution      
 Gene set null distribution of ES for **GOBP\_RESPIRATORY\_ELECTRON\_TRANSPORT\_CHAIN**
